# Effects of Environmental Enrichment on the Behavior of *Octopus vulgaris* in a Recirculating Aquaculture System

**DOI:** 10.3390/ani13111862

**Published:** 2023-06-02

**Authors:** Antonio Casalini, Laura Gentile, Pietro Emmanuele, Riccardo Brusa, Alberto Elmi, Albamaria Parmeggiani, Livio Galosi, Alessandra Roncarati, Oliviero Mordenti

**Affiliations:** 1 Department of Veterinary Medical Sciences—DIMEVET, University of Bologna, Via Tolara di Sopra 50, Ozzano dell’Emilia, 40064 Bologna, Italy; antonio.casalini6@unibo.it (A.C.); pietro.emmanuele@unibo.it (P.E.); riccardo.brusa@studio.unibo.it (R.B.); alberto.elmi2@unibo.it (A.E.); albamari.parmeggiani@unibo.it (A.P.); oliviero.mordenti@unibo.it (O.M.); 2 School of Biosciences and Veterinary Medicine, University of Camerino, Via Circonvallazione 93/95, 62024 Matelica, Italy; livio.galosi@unicam.it (L.G.); alessandra.roncarati@unicam.it (A.R.)

**Keywords:** common octopus, rearing environment, body pattern, animal welfare, cephalopods

## Abstract

**Simple Summary:**

Octopuses, like other cephalopods, have specific behaviors that correspond to a sequence of body patterns. Each pattern is the result of various components that, when performed simultaneously, have different outcomes. The vast repertoire in pattern production is associated with the complexity and variety of environmental enrichment. The greater the complexity of the environment, the greater the possibility of observing a wide variety of patterns. In this study, we evaluated how different environmental conditions affect subjects of *Octopus vulgaris* maintained in an aquaculture system through the observation of major body patterns. The results showed that octopuses kept in an enriched environment showed significantly more body patterns and gained significantly more weight than the subjects kept in a basic environment. The body patterns manifested by the octopuses maintained in a basic environment were similar to those exhibited under situations of hostility and inter/intra-specific conflict. They did not interact much with the surrounding habitat, the conspecifics, or the operator. Therefore, environmental enrichment is recommended for the individuals of this species that are kept in a recirculating aquaculture system (RAS).

**Abstract:**

*Octopus vulgaris* is a commercially valuable species. It is overexploited in the natural environment and is considered to be an innovative species for aquaculture. However, large-scale farming is generally designed only based on economic requirements, disregarding any form of enrichment that induces the natural behavior of aquatic species. Although many studies have shown the influence of environmental enrichment on terrestrial vertebrates, fish, and cephalopod mollusks, information on the effect of environmental enrichment on the body patterns of *O. vulgaris* is limited. Therefore, in this study, we assessed how different environmental conditions (Basic vs. Enriched) affect sub-adults of *O. vulgaris* kept in recirculation systems, through qualitative–quantitative studies of the main body patterns and their potential application in the commercial production of this species. The results indicated that octopuses kept in the enriched environment showed several body patterns and gained a significantly higher weight than those kept in the basic environment. The body patterns displayed by the individuals kept in the basic environment were similar to those exhibited under situations of hostility and inter/intra-specific conflict. Hence, the environment of octopuses needs to be enriched, especially for the large-scale production of this species.

## 1. Introduction

The common octopus, *Octopus vulgaris* (Cuvier, 1797), is a commercially valuable species around the world [1]. Its demand in recent years has greatly increased as it is used in various culinary preparations [2]. Individuals are captured via industrial and artisanal fishing [3] and have been overexploited in recent decades. Therefore, some countries in the European Union [4,5] have introduced strict regulations, and many researchers have investigated aquaculture techniques as an alternative source of supply [1]. In recent decades, *O. vulgaris* has been considered an innovative species for aquaculture due to its short life cycle, high growth and fertility rate, favorable food conversion index, easy adaptation to captivity, and acceptance of food of low commercial value [6,7,8]. Today, octopuses are reared in sea cages and semi-open systems on an industrial scale in Galicia, and a few years ago the rearing cycle was successfully closed thanks to a protocol patented by the Spanish Institute of Oceanography [9,10].

Aquaculture-related production is continuously expanding due to an increase in the global demand for protein sources for human consumption [11,12]. However, the large-scale production of fish requires constantly monitored and controlled environments that, if neglected and poorly maintained, may compromise animal welfare and their survival [13,14]. Under farming conditions, the environments in which fish are generally maintained are bare and/or depleted, designed only based on economic requirements. Under such rearing conditions, the development of any favorable natural behavior of fish might be restricted [11,12,15].

Efforts to improve animal welfare and reduce the adverse effects on aquaculture are reflected in the European Directive 2010/63/EU, which also includes “live cephalopods” (nautiloids, cuttlefish, squid, and octopus) [16,17]. Improving the welfare of farmed fish (e.g., reducing stress) by applying effective management protocols can increase the productivity of aquaculture [12].

Environmental enrichment (EE) strongly affects the productivity of aquaculture. Enrichment improves the welfare of captive animals by providing access to important stimuli or by promoting activities and behavioral variety [18]. The concept of enrichment is broad and involves any technique that facilitates the biological functioning of a captive animal by modifying its environment, including the encouragement of natural behaviors [19]. Environmental enrichment can be divided into different categories, depending on the objectives to be achieved [20]. Specifically, it involves (a) physical enrichment, which includes modifications or additions to tanks, i.e., structural complexity; (b) cognitive enrichment, which involves the stimulation of sensory organs and the brain; (c) nutritional enrichment, which includes varying the type and administration method of food; (d) social enrichment, which involves contact and interactions with conspecifics; (e) occupational enrichment, which involves the reduction of physical and psychological monotony by introducing environmental variations and providing opportunities for exercising and performing the preferred behaviors [17,21,22]. Before providing enrichment, it is necessary to determine whether the animal for which it is designed requires it. Thus, understanding the physiological needs of the species, its behavioral repertoire, and sensory capabilities is necessary [20]. Additionally, the changes and/or improvements resulting from the enrichment [23] also need to be evaluated. Fish and cephalopods have dissimilar behaviors, and thus, they might need different quantities and types of enrichment. Therefore, studies on EE are constantly being updated [11,17].

Although many studies have shown the influence of EE on terrestrial vertebrates, for example, rats [24,25], chimpanzees [26], and black bears [27], and on fish, such as African catfish [28], black rockfish [29], zebrafish [30,31], chinook salmon [32], rainbow trout [33], and seabream [34] that are used in research and aquaculture, there are only a few studies that have evaluated the effects of different types of environmental enrichment on cephalopod mollusks. Among these studies, the species mainly investigated include *Sepia officinalis* [35,36], *Sepia pharaonis* [37,38], *Octopus bimaculoides* [39,40], *Callistoctopus aspilosomatis* [41], *Enteropus octdofleini* [42], and *Octopus maya* [43]; however, similar studies on the behavior of *O. vulgaris* are lacking. The focus of papers on this species has been more on individual learning [44,45], social behavior [46,47], feeding behavior [48,49], problem solving [50], and play and puzzle solving [51,52,53,54]. Octopuses, like other cephalopods, show specific behaviors that correspond to a sequence of body patterns. Each of these patterns results from various components (postural, locomotor, textural, and chromatic). When these patterns and behaviors occur simultaneously, they are used to camouflage themselves in the environment, communicate with conspecifics, intimidate potential predators, and procure food. The vast repertoire of produced patterns is related to the complexity and variety of environmental enrichment [55,56]. Thus, high environmental complexity corresponds to a greater number of behaviors [57,58]. Octopuses exhibit vision-dependent intelligent behaviors, such as spatial learning, associative learning, and observational learning [41,45,55]. These characteristics suggest that octopuses are visually and tactically influenced by their surroundings, and thus, EE can strongly influence cephalopods [41]. Specifically, EE induces adult neurogenesis in the learning and multisensory integration centers, increasing cell proliferation and synaptogenesis in *O. vulgaris* [59]. Hence, behavioral observations are an effective, non-invasive indicator of welfare and an early warning system in aquaculture [60,61].

In this study, we assessed how different conditions of environmental enrichment affect the behavior of *O. vulgaris* subjects maintained in RAS and their application in the commercial production of this species.

## 2. Materials and Methods

### 2.1. Animals

Wild sub-adults of *O. vulgaris* (≥700 g body weight—BW), suitable for human consumption, were caught at the end of winter 2022 by professional fishermen, using a traditional non-invasive capture system (“polpara”), in the Ionian Sea (Gallipoli, Italy). Each animal was placed in a PVC cylinder, which was netted to avoid aggression, and transported in an insulated tank (300 L) to the laboratory in Cesenatico, where they were classified by weight and sex. The weight of each animal was recorded using an electronic scale (model WLC 20/A2, ±0.1 g, RADWAG, Radom, Poland) and then the average weight was calculated for males and females. The males were confirmed by inspecting the hectocotylus.

In total, 12 subjects were selected: 6 M, 752.7 ± 39.5 g BW, and 6 F, 723.7 ± 275.4 g BW. The animals were divided by sex into two tanks (700 L) connected to a water recirculation system. The individuals were acclimatized for three days. In this system, the initial seawater temperature (15 ± 0.5 °C, salinity 35 psu) and photoperiod (10 h light:14 h dark) matched the octopus catch conditions. After acclimatization, six couples were formed and placed in the RAS (three couples/tank).

### 2.2. Recirculating Aquaculture System (RAS)

The RAS consisted of two identical rectangular tanks (3 × 0.62 × 0.50 m; total volume 2 m^3^), a protein skimmer (0.05 m^3^), a biological filter (0.21 m^3^), and a circulation pump (maximum flow rate: 16,000 L/h). The system was also equipped with a thermal control system, a UV-sterilizing lamp, an ozonator, and an aerator.

Each tank was modified to create two types of environments and adapted to house the individuals under study. Compartments were created in both tanks with a removable grid to separate the broodstock. This grid had an opening of 20 mm, which allowed interaction between individuals while safeguarding the territorial instincts and safety of the animals. Each animal had a space of 216 cm^3^ (72 × 50 × 60) [62]. The tanks were equipped with transparent glass covers to maintain natural light conditions and prevent the animals from escaping.

### 2.3. Experimental Design

To observe the effects of environmental enrichment, two different environments were set up:

- Basic (BAS), consisting of a blue, factory-like environment, with only social enrichment (contact with conspecifics and operator) and food (ad libitum feeding and live food);
- Enriched (ENR) environment, with the presence of physical enrichment: substrate (sand), wall color (the walls were “naturalized” with beige-colored polypropylene panels); cognitive enrichment: the presence of seashells, stones, and plastic toys; social enrichment (contact with conspecifics and the operator) and food (ad libitum feeding and live food).

In total, six individuals (3 M and 3 F) were placed in the two different environments for a total of six replicas in each condition. Each replica was placed in the tank and had its own artificial den.

For behavioral observations, we followed the body patterns and components reviewed by Borrelli et al. [63]. Observations were made for 4 h/day (9–11 a.m. and 3–5 p.m.), 5 days per week, and were performed by previously trained staff. For data collection, a special form was developed where the staff member could make daily records including the subject, the number of occurrences of behaviors, and add any additional comments. All daily observations were then transferred to the computer and processed as follows:

- the total number of observations in the two environments;
- the total number of observations in both sexes (regardless of the environment);
- the number of weekly observations in the two environments;
- the percentage of observations of various behaviors in each environment.

All octopuses underwent the conditioning program, which consisted of an increase in temperature of 1 °C/week until 20 °C and an increase in a photoperiod of 1 h/month up to 15 h light and 9 h dark [64]. The animals were fed ad libitum once a day with a mix of frozen (20%) and fresh (80%) fish and crustacean (40% *Squilla mantis*, 40% *Carcinus* sp., and 20% *Boops boops*) [64].

### 2.4. Growth Performance

All animals were weighed at the beginning (Wi, initial weight in g), every two weeks, and at the end of the experiment (Wf, final weight in g). The following indices were calculated:

Specific Growth Rate (SGR) (% BW/day) =

$$\left[\left(\ln\mathrm{Wf}-\ln\mathrm{Wi}\right)÷\mathrm{Days}\right]\times 100$$

Absolute Growth Rate (AGR) (g/day) =

$$\left(\mathrm{Wf}-\mathrm{Wi}\right)÷\mathrm{Days}$$

### 2.5. Statistical Analyses

To test the behavioral differences between individuals in the two environments, PERMANOVA was conducted based on the environmental factor (two levels: Basic and Enriched) and the sex factor (two levels: male and female). To find differences between individuals from the two environments, the normality of the data was first checked by performing Shapiro–Wilk tests. Since the data did not follow a normal distribution, non-parametric tests (Wilcoxon tests) were conducted for each behavior, considering the environmental factor. A two-way ANOVA for all the individuals was conducted considering the factors (time and environment) and the variable weight. Another two-way ANOVA was performed on the variable number of behaviors considering the environmental condition and temporal factors. First, normality was determined by the Shapiro–Wilk test, and the homogeneity of variances was determined by Cochran’s test (*p* > 0.05). Tukey’s post hoc test was performed after the ANOVA.

Finally, the variables SGR and AGR were also tested by performing ANOVA, considering the environment as a factor. For both variables, preliminary tests were conducted to determine the normality and homogeneity of variances.

All ANOVA and Wilcoxon tests were performed using R (R Development Core Team; packages “GAD”, “ggplot”, “fmsb” 2018). The PERMANOVA test was performed using Past 4.10.

### 2.6. Ethics

All octopuses were handled following the regulations of the European Union concerning the protection of experimental animals (Dir. 2010/63/EU) and the regulations of the Ethics Committee of Bologna University (prot. ID 4459-17 February 2023).

## 3. Results

**Figure 1:**
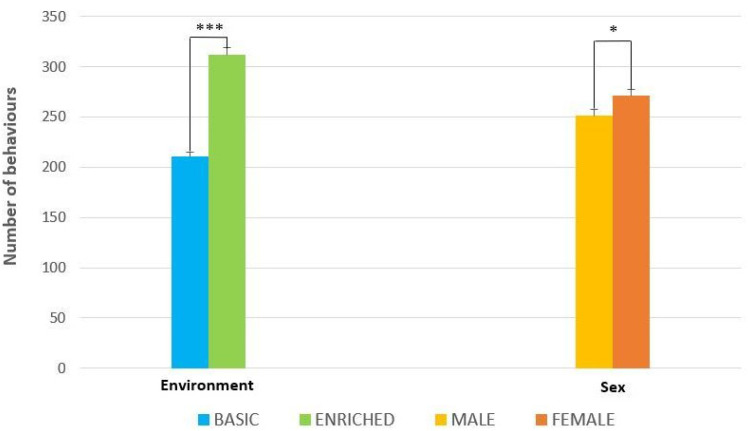
The effect of environment and sex on the total number of observations. The data are shown as mean ± standard deviation. Asterisks represent statistically significant differences between the environments (Basic vs. Enriched) and sex (male vs. female) (PERMANOVA; *** *p* < 0.01 and * *p* < 0.05).

**Figure 2:**
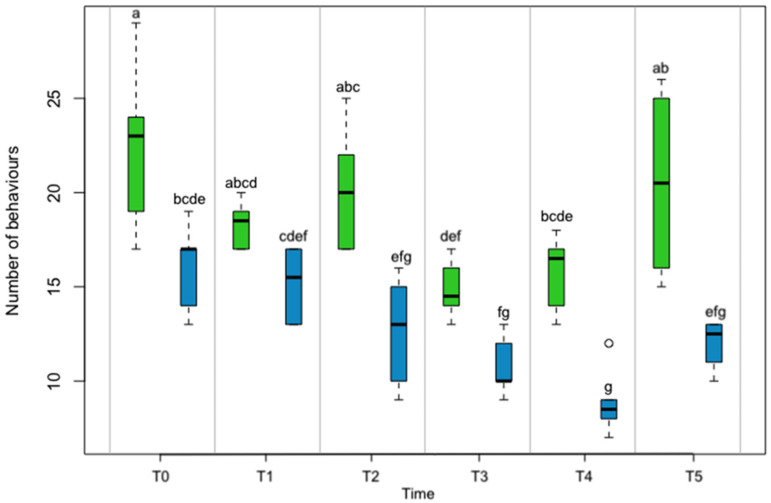
Boxplot showing the trend in the total number of behaviors in the Enriched (green) and Basic (blue) environments at various time intervals. Significant differences between the environmental conditions at each experimental time are marked by dissimilar letters (a, b, c, d, e, f, and g) (*p* < 0.05).

The study was conducted for six weeks, during which, 14 body patterns and 5 components were identified in the ENR tank, whereas 9 body patterns and 3 components were identified in the BAS tank (Table 1). Specifically, the environmental factor significantly (*p* < 0.001) influenced the total number of behavioral observations (body patterns and components) (311.6 ± 0.6 ENR vs. 210.4 ± 0.7 BAS) (Figure 1). The expression of body patterns was also significantly influenced by the sex factor (*p* < 0.05) (Figure 1). However, these variables were not influenced by the interaction between the sex factor and the environmental factor (*p* = 0.1967). The results of ANOVA showed that both factors significantly affected the number of observations (*p* < 0.001), but the interaction between the factors was not significant (Figure 2). The octopuses in the ENR tank exhibited a higher number of behaviors in the first week than the octopuses in the BAS tank, a trend that remained unchanged throughout the trial. In contrast, the octopuses in the BAS tank showed a steady decrease in the number of behaviors from the first week to the fifth week and then increased the number of behaviors in the last week of observations. From the results of Tukey’s test, among the time levels, the comparison between T1 and T5 and T3 and T4 was not significant. When comparing the two environments across time levels, all time intervals except T1 and T3 were significant. Within ENR, the comparison between T3 and T2 was significant (*p* < 0.05) (Figure 2).

**Figure 3:**
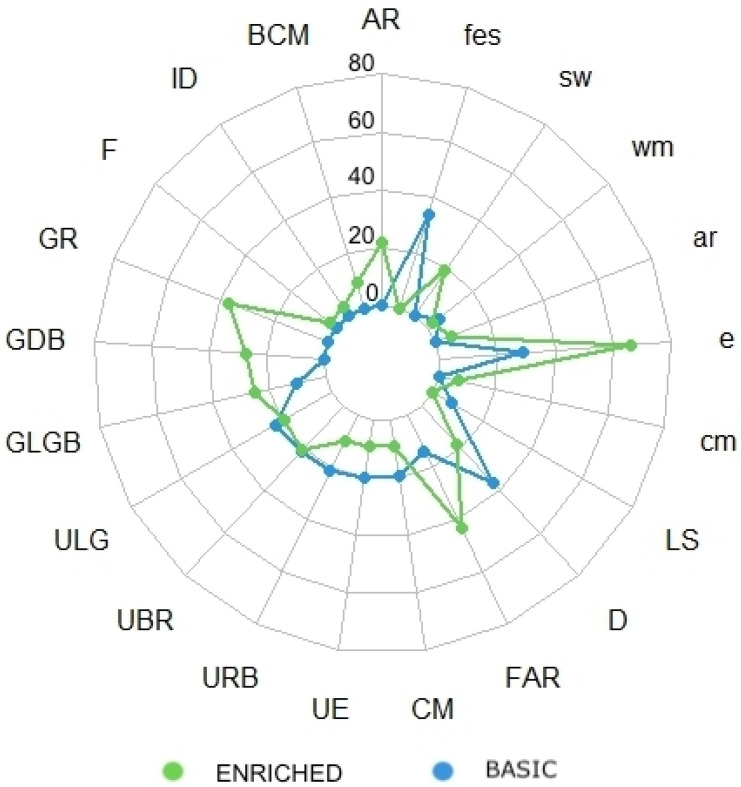
A radar chart representing total body patterns and components exhibited in the two environments (Basic and Enriched); *p* < 0.05 for all behaviors/components exhibited, except for *UBR*, *ULG, LS*, and *wm*.

Regarding the influence of the environment on each body pattern and manifested component, the Wilcoxon test showed significance for all recorded behaviors/components with *p* < 0.05 except for Uniform Brownish-Red (*UBR*), Uniform Light Gray (*ULG*), Longitudinal Stripes (*LS*), and Withdrawal maneuver (*wm*) (Figure 3).

In the BAS environment, the most prevalent patterns included *ULG* (19.7 ± 0.9), *UBR* (18.7 ± 1.2), *URB* (18.7 ± 1.1), *CM* (17 ± 0.8), and *D* (32 ± 1.9). Among the components, *e* (25.3 ± 1.4) and *fes* (29.7 ± 1.6) were the most prevalent (Figure 2).

In the ENR environment, the most prevalent patterns included *GR* (32.3 ± 1.1), *GDB* (24 ± 1), *GLGB* (22 ± 1.2), *AR* (19 ± 0.8), and *FAR* (38 ± 1.6). The most prevalent components included *e* (58 ± 0.4), *cm* (6 ± 0.6), and *sw* (16.3 ± 0.8) (Figure 2).

**Figure 4:**
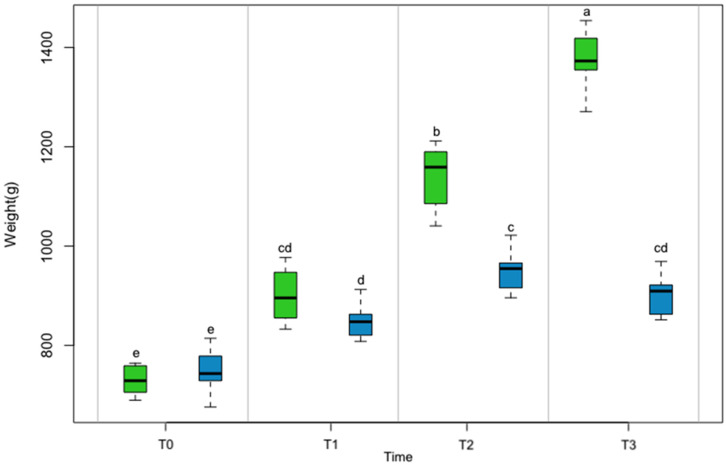
Boxplot showing the weight gain of octopuses in the Enriched (green) and Basic (blue) environments at various time intervals. Significant differences among environmental conditions at each experimental time point are shown using dissimilar letters (a, b, c, d, and e) (*p* < 0.05).

When the weight of the animals was analyzed without differentiation based on sex, the two-way ANOVA showed that the factors of environment and time significantly influenced (*p* < 0.001) the weight variable (Figure 2). The octopuses in the ENR environment gained significantly more weight at the end of the test than those in the BAS environment (1373.9 ± 62.3 g vs. 903.8 ± 42.5 g) (Table 2). The post hoc test for the BAS environment factor showed that all comparisons for the different time level intervals were significant except for those between T3 and T1 and T3 and T2. In the ENR environment, all comparisons across time levels intervals were highly significant (*p* < 0.01). In the comparison between the two levels of the environment in the various time levels, comparisons between T1 and T0 between ENR and BAS were not significant (Figure 4).

The results regarding SGR and AGR showed that for both indices, the environment had a statistically significant influence (*p* < 0.01). Significantly higher values (SGR = 51 ± 0.1% and AGR = 15.4 ± 1.2 g) were found in the ENR environment than in the BAS environment (0.5 ± 0.1% and 3.7 ± 0.6 g) (Table 2).

## 4. Discussion

Enrichment in aquaculture can be defined as the addition of new environmental stimuli to meet the physiological and behavioral needs of the farmed species. Tonkins et al. [35] and Arechavala-Lopez et al. [11] showed that several aquatic species, when maintained in enriched environments, can improve their behavior and, consequently, their welfare.

In this study, we found that in the common octopus kept in captivity, different environmental conditions can influence its behavior during the growing phase. We also found that enrichment conditions (ENR) positively affected its behavior and welfare. Under ENR conditions, the octopuses showed increased activity and interaction, both intra-specifically and inter-specifically and the most frequent behaviors were those that indicated a calm condition. Specifically, the welfare manifestation of ENR octopuses was reinforced by the high number of observations of body patterns related to ‘camouflage’ (*GR*, *GDB*, *ULG*, and *GLGB)* that, according to Hanlon and Messenger [55], Borrelli et al. [63], and Cowdry [65], is a key manifestation of octopus well-being.

Although the main body patterns associated with intraspecific and interspecific conflicts and/or threats (*CM*, *EU*, *UBR*, and *URB*) were recorded in both environments, there were substantial differences in the quantity and time spent performing these patterns. Specifically, in the ENR environment, they were exhibited for a short period, generally at the beginning of the trial, while in the BAS environment, they were exhibited almost constantly throughout the trial. In the BAS environment, the presence of social and dietary enrichment alone was not sufficient for ensuring a good fit of octopuses, although they are known to have a beneficial effect on the behavior of other aquatic species, especially those used in farming [11]. Some patterns had different values in two different environments; for example, *D* had a positive valence in the octopuses maintained in the ENR, and it was recorded only during resting, denning, and food consumption, while in the BAS, it was frequently exhibited to avoid contact with the surrounding environment. The greater difficulty in adapting to the BAS environment was also indicated by the locomotor component, which was abandoned by octopuses in the BAS by mid-trial. These individuals exhibited hostile behaviors toward conspecifics and external sources (operators), and their ability to change patterns during the entire trial was limited. The result regarding dynamic responses was unexpected. This is a disturbance behavior that occurs when the animal is in danger, unprepared, and outside its den [56]. This behavior was exhibited in a few individuals and to a limited extent (1.5%) only in ENR octopuses and in an incomplete form (Incomplete Dymantic, *ID*), i.e., when the animal was in the den. This behavior might be related to the “personality” of the animal, i.e., variations in behaviors between individuals determined by genetic and environmental factors [69,70]. A study on *Octopus rubescens* suggested that octopuses might have personalities like other animals [69]. Given that the *ID* pattern was infrequent, we hypothesized that this might be a fear reaction related to the initial mistrust of some individuals toward operators, which was gradually overcome. However, Yasumuro and Ikeda [41] studied *C. aspilosomatis* and found that this pattern was exhibited by octopuses in the bare tank with significantly greater frequency than in their counterparts who were maintained in the enriched environment.

The color of the tank associated with the depth, source of light, and clarity of the water affects the degree to which light is absorbed, reflected, diffused, and attenuated in the farming environment. Thus, in the case of *O. vulgaris*, a species that has excellent vision and continuously searches for camouflage in the environment [71], the color of the tank is an essential aspect of the aquaculture sector.

In this study, the blue background of the BAS tank may have been the cause of different behaviors among individuals. The important role of color in cephalopods was also highlighted by Okamoto et al. [72], who observed that adults of *O. vulgaris* and *O. aegina* preferred black, red, and orange tanks when offered a choice among seven different colors. McLean [73] also found that tank and background color of commercial breeding tanks, which are mainly produced in blue, black, or green, can affect the physiological and behavioral processes of teleosts, elasmobranchs, amphibians, and marine invertebrates, including cephalopods. Batzina et al. [74,75] and Batzina and Karakatsouli [76] showed that individuals of *Sparus aurata* (giltheads) maintained in a controlled environment preferred a blue substrate and showed less aggressive behavior. In contrast, *O. niloticus* and *P. trituberculatus* showed higher growth and better growth indices in blue-colored tanks [77,78]. The color of the tank has also been shown to influence reproduction rates; in fact, Volpato et al. [79] found a higher reproductive rate associated with increased excavation and construction of the nest in Nile tilapia maintained in tanks with a blue substrate.

Both physical enrichment (sand) and cognitive enrichment (objects of various kinds) improved the lives of the octopuses in the tank, considering that they exhibited the behaviors that are normally performed by this species in a natural environment using visual and tactile cues. Many studies have shown that the presence of a substrate, such as sand, can improve the welfare of aquatic species, especially those that interact with the bottom or live close to it [11,21].

In a study, *S. officinalis* showed better behavior when kept in tanks containing sand than in those without it [35]. Additionally, since cuttlefish perform sand digging for long durations until they attain sexual maturity [80], environmental limitations might affect their development and cognitive learning [55]. Flatfish also benefit from the presence of substrate in rearing tanks and exhibit increased resting behavior, fewer skin lesions, and increased growth [81,82].

Not only the presence of a sandy bottom but also the presence of objects, such as seashells, stones, and plastic toys, in the ENR tank played a positive role as cephalopods possess high cognitive abilities and need a continuous motor and cognitive stimulation [17,83]. This ability to interact with objects has been found in *Octopus maya* under laboratory conditions [43] and also in *Octopus vulgaris* in the wild [51]. Cuttlefish farmed in environments with objects, such as artificial algae and rocks, showed normal development of learning and memory, unlike those in unenriched environments [36]. Beigel and Boal [40] and Yasumuro and Ikeda [41] found that individuals of *O. bimaculoides* and *C. aspilsomatis* farmed in tanks with some objects/toys were more active and stimulated than those kept in tanks that lacked them. This correlation, important in aquaculture, has also been observed in other fish species. Zhang et al. [29] studied black rockfish, Ojelade et al. [28] studied African Catfish, Batzina and Karakatsouli [84] studied gilthead seabream, and Rosengren et al. [85] studied Atlantic Salmon and found a greater weight gain in fish exposed to physical enrichment conditions than those reared under sterile conditions.

Regarding zootechnical performance, ENR octopuses showed a greater weight gain than BAS ones. Additionally, the AGR and SGR values in the ENR tank showed three times higher values than the BAS tank, and these results correspond to those of other studies in which a suitable environment and a predominantly crustacean diet were found to promote octopus growth [64,86,87]. In contrast, octopuses kept in BAS tanks showed inappetence and rejection of food, which led to a decrease in their weight. These results are also supported by the different behaviors expressed by the octopuses., e.g., certain patterns such as the FAR (behavior performed during a prey attack), which was exhibited more in the ENR environment and was directed exclusively towards the administered food (live crustaceans), while in the BAS tank, it was used by the octopuses mainly as a hostile behavior towards conspecifics, in particular by males towards females, followed by the locomotor defensive component (removal maneuver). Low growth is a consequence of fatigue caused by living in a basic environment. Chronic difficulties have inhibitory effects on growth and energy metabolism in farm animals [88]. In a study conducted on *S. pharaonis* [38], individuals maintained in a bare environment lacking any enrichment showed lower growth and reduced behavioral abilities than those maintained in an enriched environment.

The limited number of body patterns associated with the onset of weight loss recorded in octopuses maintained in the BAS environment like an increase in denning *(D*), led to the decision to suspend the trial to ensure the welfare of the animals.

From the perspective of commercially farming this species, this study highlighted that *O. vulgaris* maintained in captivity needs adequate environmental enrichment. Breeding in a bare, noisy, and chromatically unsuitable environment, such as many recirculation systems (RAS), not only reduces the zootechnical performance of this species but also might endanger its survival. However, the issues related to the increased cost of environmental enrichment [22] and the increased sanitation issues that might occur in a closed-loop system due to the presence of extraneous elements and sandy substrates in the tank should not be ignored.

## 5. Conclusions

The results obtained in this work highlighted how octopuses, kept in RAS, require an enrichment as close as possible to the natural one (ENR). In the ENR environment, the octopuses exhibited significantly more patterns/components and behavioral observations demonstrating how an enriched environment improves social interactions and promotes greater weight gain than BAS subjects, an aspect not to be underestimated especially in view of the commercial breeding of the species.

## Figures and Tables

**Table 1:** Body patterns and components identified in different environments (Basic and Enriched). The frequency of observation is reported as a percentage. The ✓ means presence, ✗ means absence.

N	Body Pattern	Meaning	BAS	ENR	BAS%	ENR%	References
**1**	Conflict Mottle—*CM*	Disturbance	✓	✓	11.3	3.4	[56,65]
**2**	Unilateral Effect—*UE*	Disturbance	✓	✓	11.5	3.4	[56]
**3**	Uniform Brownish- Red—*UBR*	Intraspecific encounters, disturbance	✓	✓	12.4	8.2	[65]
**4**	Uniform Reddish- Brown—*URB*	Social interactions, disturbance	✓	✓	12.4	3.9	[65]
**5**	Full Attack Response—*FAR*	Feeding behavior	✓	✓	7.9	16.9	[63]
**6**	Denning—*D*	Rest, feeding, disturbance	✓	✓	21.2	7.1	[55]
**7**	Uniform Light Gray—*ULG*	Camouflage	✓	✓	13.0	7.4	[65]
**8**	Ground Light Grayish- brown—*GLGB*	Camouflage, rest	✓	✓	6.0	9.8	[65]
**9**	Longitudinal Stripes—*LS*	Intraspecific interactions, disturbance	✓	✗	4.3	-	[56,65]
**10**	Fighting—*F*	Intraspecific interactions	✗	✓	-	1.2	[63]
**11**	Incomplete Dymantic—*ID*	Disturbance	✗	✓	-	1.5	[56]
**12**	Broad Conflict Mottle—*BCM*	Disturbance	✗	✓	-	3.6	[66]
**13**	Acute Resemblance—*AR*	Camouflage	✗	✓	-	8.5	[67]
**14**	Ground Dark Brown—*GDB*	Camouflage, rest	✗	✓	-	10.7	[65]
**15**	General Resemblance—*GR*	Camouflage	✗	✓	-	14.4	[63,67]
**N**	**Components**	**Meaning**	**BAS**	**ENR**	**BAS%**	**ENR%**	**References**
**1**	Envelope—*e*	Prey capture	✓	✓	42.7	66.4	[63]
**2**	Withdrawal Manoeuvre—*wm*	Defensive posture	✓	✓	7.3	2.3	[56]
**3**	Funnel Directed Toward External Stimulus—*fes*	Reaction against the disturbance	✓	✗	50	-	[56]
**4**	Cleaning Manoeuvre—*cm*	Rapid twirling of the arms	✗	✓	-	6.9	[56]
**5**	Arms Raised—*ar*	Postural component	✗	✓	-	5.7	[68]
**6**	Swimming—*sw*	Locomotor component	✗	✓	-	18.7	[66]

**Table 2:** The growth trend of *O. vulgaris* subjects in the Enriched and Basic tanks. Shown below are the initial weight (Wi, g), final weight (Wf, g), Specific Growth Rate (SGR, %), and Absolute Growth Rate (AGR, g/d). The data are presented as the mean ± S.D. Asterisks represent statistically significant differences between the environments (** *p* < 0.01).

	Animal	Wi (g)	Wf (g)	SGR (%)	AGR (g/d)
**ENR**	EF1	731.7	1354.8	1.47	14.8
	EF2	689.3	1270.8	1.46	13.8
	EF3	764.1	1371.4	1.39	14.5
	Mean F	728.4 ± 37.5	1332. 3 ± 53.9	1.44 ± 0.04	14.4 ± 0.5
	EM1	705.4	1374.1	1.59	15.9
	EM2	758.8	1418.4	1.49	15.7
	EM3	725.7	1454.2	1.65	17.3
	Mean M	730 ± 27	1415.6 ± 40.1	1.58 ± 0.1	16.3 ± 0.9
	**MEAN ENR**	**729.2 ± 29.2**	1373.9 ± 62.3 **	1.51 ± 0.1 **	15.4 ± 1.2 **
**BAS**	PF1	728.7	911.4	0.53	4.4
	PF2	675.6	862.7	0.58	4.5
	PF3	752.8	907.1	0.44	3.7
	Mean F	719 ± 39.5	893.7 ± 27	0.52 ± 0.1	4.2 ± 0.4
	PM1	778.3	921.4	0.40	3.4
	PM2	814.2	968.9	0.41	3.7
	PM3	733.6	851.4	0.35	2.8
	Mean M	775.4 ± 40.4	913.9 ± 59.1	0.39 ± 0.03	3.3 ± 0.4
	**MEAN BAS**	**747.2 ± 47.2**	**903.8 ± 42.5**	**0.5 ± 0.1**	**3.7 ± 0.6**

## Data Availability

The data presented in this study are available on request from the corresponding author.
